# Covalently-Bonded Coating of L-Arginine Modified Magnetic Nanoparticles with Dextran Using Co-Precipitation Method

**DOI:** 10.3390/ma15248762

**Published:** 2022-12-08

**Authors:** Behnam Azadpour, Faezeh Kashanian, Mehran Habibi-Rezaei, Seyyed Ali Seyyed Ebrahimi, Roozbeh Yazdanpanah, Zahra Lalegani, Bejan Hamawandi

**Affiliations:** 1 Advanced Magnetic Materials Research Center, School of Metallurgy and Materials, College of Engineering, University of Tehran, Tehran 111554563, Iran; 2 School of Biology, College of Science, University of Tehran, Tehran 111554563, Iran; 3 Department of Applied Physics, KTH Royal Institute of Technology, SE-106 91 Stockholm, Sweden

**Keywords:** magnetite nanoparticles, multi-functionalized nanoparticles, magnetic nanocomposties, L-arginine, dextran, co-precipitation method

## Abstract

In this study, L-arginine (Arg) modified magnetite (Fe_3_O_4_) nanoparticles (RMNPs) were firstly synthesized through a one-step co-precipitation method, and then these aminated nanoparticles (NPs) were, again, coated by pre-oxidized dextran (Dext), in which aldehyde groups (DextCHO) have been introduced on the polymer chain successfully via a strong chemical linkage. Arg, an amino acid, acts as a mediator to link the Dext to a magnetic core. The as-synthesized Arg-modified and Dext-coated arginine modified Fe_3_O_4_ NPs were characterized by scanning electron microscopy (SEM), X-ray diffraction (XRD), Fourier transformation infrared spectroscopy (FT-IR), thermogravimetric analysis (TGA), and vibrating sample magnetometer (VSM). Both synthesized samples, XRD pattern and FT-IR spectra proved that the core is magnetite. FT-IR confirmed that the chemical bonds of Arg and Dext both exist in the samples. SEM images showed that the NPs are spherical and have an acceptable distribution size, and the VSM analysis indicated the superparamagnetic behavior of samples. The saturation magnetization was decreased after Dext coating, which confirms successive coating RMNPs with Text. In addition, the TGA analysis demonstrated that the prepared magnetic nanocomposites underwent various weight loss levels, which admitted the modification of magnetic cores with Arg and further coating with Dext.

## 1. Introduction

Nowadays, nanotechnology, “which design, characterize, and utilize structures, devices, and systems by controlling their shape and size at the nanoscale,” has become one of the most interesting and applicable parts of research [1]. In this case, NPs, particles whose at least one of their dimensions is less than 100 nm [2], have been mostly noticed since the physiochemical properties of the material are influenced by its size and shape, and it can help scientists gain appropriate functions from the material by controlling them [2,3]. NPs are used for drug delivery [3], chemical and biological sensing [4], gas sensing [5], CO_2_ capturing [6], etc.

One of these NPs that show superparamagnetism behavior is magnetite (Fe_3_O_4_). Superparamagnetic NPs refer to particles with a large magnetic moment in the presence of an external magnetic field, because of the coupling of atomic spins, without showing magnetism after removing the magnetic field [7,8].

Magnetite, because of its biocompatibility, nontoxicity, and generally its FDA approval [9], is widely used for biomedical applications such as tissue repair [10], cellular therapy [11], separating and purifying cell populations [12], radioactive therapies [13], tumor hyperthermia [14] and magnetic resonance imaging (MRI) [15].

Arginine, classified as amino acids materials, has many potential biomedical applications, as it is highly biocompatible, provides several functional groups to attach to other materials for various goals, and could account for necessary components for proteins production known as one of the essential building blocks of living organisms. Arginine has a carboxylic acid, an amine, and a guanidine group, which could be protonated and deprotonated in different pHs, so that it is able for Arg to make electrostatic bonding with charged materials [16,17,18]. The aforementioned functional groups give Arg the opportunity of making chemical linkages through different mechanisms, such as the Schiff-base reaction between aldehyde groups (-CHO) and amine groups (-NH_2_), as well as what can be observed in peptide formation reaction (forming covalent bonding between carboxylic acids (-COOH) and amines (-NH_2_)). Arg, as a nanocarrier or modifying agent for NPs, could be exploited in many fields, some of which are related to dual model imaging functionalized iron oxide NPs [19], delivery of therapeutic agents, such as paclitaxel, for tumor treatment [20], delivery of nucleic acids (gene delivery) [21], oral drug delivery [22], etc.

In the previous studies carried out by our research group [23,24,25,26], L-arginine modified magnetic nanoparticles (RMNPs) were synthesized by the co-precipitation method in different concentrations of arginine to find the optimum concentration and then found the effect of these decorated NPs or bare Fe_3_O_4_ on the stability of proteins as a novel application of Fe_3_O_4_.

Despite the referred applications, due to the high surface-to-volume ratio, Fe_3_O_4_ NPs tend to aggregate because of the strong magnetic dipole interaction between particles [27], resulting in a wide distribution of particles that consequently limits its applications by lowering magnetization [28,29]. Therefore, to prevent the agglomeration of Fe_3_O_4_ NPs and induce colloidal stability, they can be functionalized via various components, including amino acids such as L-arginine [23] and L-cysteine [30], natural polymers such as trehalose [31], Dext [32], chitosan [33], synthetic polymers such as polyethylene glycol [34] or inorganic materials such as silica [7] to make a steric hindrance between particles [16].

Surface functionalization also can be used to introduce desirable functional groups on the surface of the magnetic nanoparticles (MNPs) to attach different moieties to the MNPs, such as antibodies [35] and therapeutic agents [28] for different biomedical applications, while the bare MNPs lack the conjugating capability.

Dext is a polysaccharide, classified as natural polymer. It is widely used to prepare polymeric NPs [36], hydrogels [37], microgels [38], and nanogels [39] and is used to functionalize the MNPs for biomedical applications [8]. Dext is a biocompatible long-chain hydrophilic polymer that could physically adsorb on MNPs via non–covalent interactions [27]. This coating is very popular because of its properties such as solubility in water, biocompatibility, and effective stabilization of colloidal forms due to electrostatic repulsion [40]. Much research work has been performed in coating MNPs with Dext. For example, Shaterabadi et al. [41] prepared biocompatible dextran-coated magnetic NPs in various Dext concentrations. They found that the size of the as-synthesized NPs were reduced with increasing Dext content, showing that Dext has a surfactant effect that is potent to be used for further theranostic applications due to its biocompatibility. In another research work [42], Dext has been used as a biocompatible coating to load curcumin, which is challenging to administer in the body due to its hydrophobic nature for antimicrobial targets. Predoi research group [43] synthesized Dext-coated magnetite NPs. Afterward, they deposited a thin layer of synthesized NPs on a glass substrate to investigate using these biocompatible NPs as a coating layer for biomedical devices. Despite all, the bond between NPs and Dext is hydrogen bonding, which is weaker than other types of linkages and consequently leads to dissociating the Dext from its core in harsh and stressed environments such as biological media. To address this issue, Dext must undergo various modification processes to be endowed with unique properties.

Several hydroxyl groups, with existence on the main chain, make Dext highly soluble in water, candidate it to graft with various functional groups and components for drug delivery for further interactions with biological molecules such as proteins, enzymes, antibodies, etc. Some hydroxyl groups can graft to hydrophobic components, such as cholesterol, to make Dext amphiphilic. It is possible to modify Dext with aldehyde groups for further conjugation using oxidative agents such as periodate. Charged derivatives of Dext are also obtainable via introducing carboxylic acid and amine groups, which gives Dext a negative and positive electrical charge, respectively [44].

Several scientists have worked on coating the magnetite with a layer of Dext, demonstrating that it promotes magnetite’s stability during blood circulation [45,46]. Dext-MNPs are widely used as a contrast agent in MRI [47] and magnetically mediated hyperthermia [27]. Dext was bonded to bare MNPs through weak van der Waals forces and hydrogen bonds, and it may be desorbed from the surface of magnetite in the aqueous media such as blood. Therefore, Dext must be bonded to magnetite through a strong chemical linkage to solve this problem [48].

This article aims to attach the pre-oxidized aldehyded dextran (DextCHO) to the MNPs via a chemical linkage, which is our research’s main point, focus, and novelty. There have been some studies that focus on the chemical attachment of Dext to the MNPs with several mediator materials such as 3-aminopropyltriethoxysilane (APTES) [48], but to the best of our knowledge, it is for the first time that Arg has been used as a mediator to attach Dext to the MNPs chemically. For this purpose, modified MNPs were first synthesized using Arg as a modifying agent. Afterward, DextCHO was bonded to the RMNPs by forming C–N chemical bonds between the aldehyde groups of DextCHO and the amine groups of Arg. These newly synthesized magnetic nanocomposites could be potent in their use as a nanocarrier for drug delivery, contrast agents for MRI, and measuring its effect on protein denaturation and structural changes.

## 2. Experimental

### 2.1. Materials and Instrumentation

Iron (Ⅲ) chloride hexahydrate (FeCl_3_.6H_2_O), iron (Ⅱ) chloride tetrahydrate (FeCl_2_.4H_2_O), Arg, sodium borohydride (NaBH_4_), sodium periodate (NaIO_4_), ammonia solution (25 wt.%) and hydrochloric acid fuming (37 wt.%) were all purchased from Merck (Darmstadt, Germany). Dext with an average molecular weight of 40 kDa was prepared from Sigma-Aldrich. During the whole steps of the synthesis procedure, deionized water (DI water) is used.

The XRD technique was used to determine the crystalline structure of the synthesized NPs. MNPs were characterized by XRD using a Rigaku-Dmax 2500 diffractometer, with Cu K_α_ radiation (λ = 1.5406 °A) and 2 theta from 5 to 90° and the warming rate of 0.02 °min^−1^. The magnetic properties of RMNPs and Dext-RMNPs samples at room temperature were measured by a VSM (Meghnatis Daghigh Kavir Co., Kashan, Iran). Fourier transforms infrared (FTIR, Rayleigh WQF-510A, Beijing, China) spectra of the two synthesized MNPs (RMNPs and Dext-RMNPs), arginine, and DextCHO in powder form were recorded using KBr pellets spectrophotometer. FTIR spectra were obtained in the range of 400–4000 cm^−1^ to approve the formation of Arg and Dext coating around the magnetic core and the detection of the other formed chemical bonds. RMNPs and DextCHO-SEM measured RMNPs size and morphological structure with 30,000 accelerating voltage and magnifications of 40,000, 80,000 and 160,000 made by the FEI Company of USA (model of Quanta 200). For this purpose, RMNPs and DextCHO-RMNPs powders were suspended in distilled water, sonicated, and dropped on the surface of the silicon wafer. The samples were then subjected to gold sputtering, which led to coating a layer of gold on the samples. This process has been conducted to make the samples conductive and avoid electrical charge accumulation. The average particle size was obtained using the ImageJ tool on SEM images. The measurements were performed on more than 80 particles. RMNPs and DextCHO-RMNPs were subjected to thermogravimetric analysis using a thermogram (TGA/DSC 3+, Mettler Toledo Co., Colombus, OH, USA) to find the coating efficiency. For this purpose, 5 g of each sample was placed on an alumina sample holder with an initial temperature of 25 °C, and the nitrogen gas flowed with a flow rate of 50 mL min^−1^ and a heating rate of 10 °C min^−1^. The heating process was continued to 800 °C, and the weight loss was measured as a function of temperature.

### 2.2. Dextran Oxidation

Dext was oxidized utilizing sodium meta periodate (NaIO_4_), as a strong oxidizing agent to attack the glucose rings in the chain of the Dext, break C–C bonds, and produce aldehyde groups on the polymer (DextCHO). According to the protocol by Su et al. [38], 5 g of the Dext was added to 40 mL of DI water and stirred for 10 min to completely dissolve in the water. After that, 6.6 g of NaIO_4_ was mixed with a prepared polymeric solution and stirred for 6 h in the darkness. To finish the oxidation reaction, a 2.1 mL glycerol was added to the oxidized Dext solution, which has a color between yellow and orange. To remove the IO_4_^1−^ ions, the solution was dialyzed against distilled water for three days in a dialysis bag with 14 kilodaltons cut off. As the purification was preceding, the color of the solution faded. Finally, the colorless solution was brought for freeze drying to attain the solid material, which was further used to characterize and coat the RMNPs.

### 2.3. Synthesis of RMNPs

To produce the RMNPs, firstly, FeCl_3_.6H_2_O and FeCl_2_.4H_2_O were mixed with DI water in the molar ratio of 2:1. Simultaneously, Arg solution with a concentration of 1 mM was prepared and poured into salt solution drop by drop under vigorous stirring and nitrogen atmosphere at room temperature. After 1 h, the synthesis procedure of RMNPs was completed by adding ammonia solution (25%) and obtaining black precipitates of RMNPs. These precipitates were separated from the suspension by a magnet, washed three times with DI water to remove Arg residue and solvent, and dried under a vacuum at 40 °C.

### 2.4. Preparation of DextCHO-RMNPs

At first, 0.25 g of RMNPs were dissolved in 20 cc DI water. Then, the suspension was probe sonicated for 15 min to remove the aggregates and obtain a uniform and monodispersed suspension. At the same time, 100 mg of DextCHO was dissolved in 35 mL of DI water and after that pH of the prepared solution was adjusted to 3 by adding hydrochloric acid fuming (37%). Acidified DextCHO solution was added to NPs’ suspension drop by drop for 10 min under nitrogen purging, and then the temperature should reach 60 °C and is stirred for 24 h. After that, the ammonia solution was added. Later, the suspension’s pH must reach 9 by adding ammonia solution and stirring for 4 h at a dark place. Finally, 0.6 g of sodium borohydride (NaBH_4_) was added to the suspension and stirred for 1 h. At last, DextCHO-RMNPs were separated by a magnet from suspension, washed with DI water and ethanol several times, and dried at 40 °C for 24 h.

## 3. Results and Discussion

**Figure 1:**
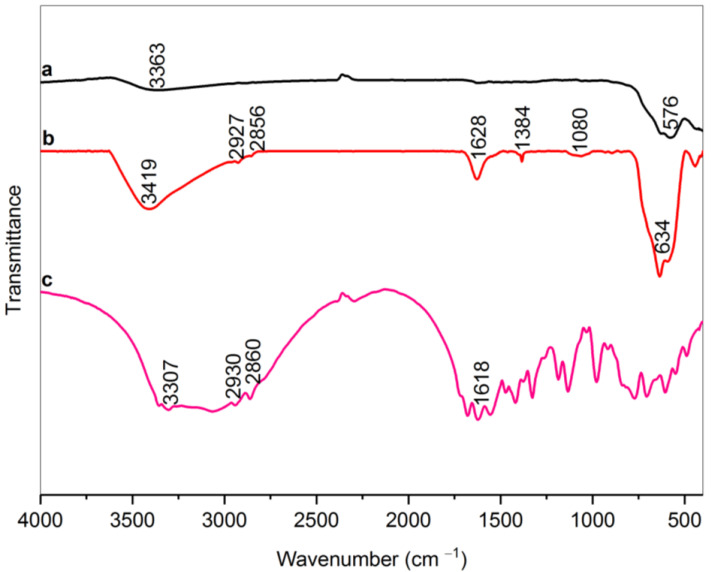
FT-IR spectra of bare MNPs (a), RMNPs (b), and Arg (c).

The FTIR spectra of bare MNPs, RMNPs, and pure arginine are depicted in Figure 1. A characteristic absorption peak in the range of 570–630 cm^−1^ in samples MNPs and RMNPs are attributed to Fe–O bond vibrations. The weak and broad peak at 3363 cm^−1^ in the MNPs is related to stretching vibrations of the O–H bond that can be associated with the adsorbed water molecules on the MNPs’ surface [23,48]. In the modified arginine magnetite NPs sample spectrum, the peak at the 1080 cm^−1^, which is due to C–N stretching vibration, 1384 cm^−1^, 2856 cm^−1^, and 2927 cm^−1^ are attributed to scissoring, symmetric, and asymmetric vibration C–H bonds in methylene groups (CH_2_); 1628 cm^−1^ and 3419 cm^−1^ that is caused from N–H bending and stretching vibrations relatively in NH_2_ terminals of arginine [49]. All of these indicate that magnetite NPs were successfully modified with arginine. It is necessary to state that the absence of bands in the ranges of 1210–1320 cm^−1^ and 1700–1730 cm^−1^, which correspond to the C–O stretching and C=O stretching vibration, respectively [50], can be attributed to the fact that carboxylic acid parts of the Arg were not free, and were probably reacted with the OH groups on the surface of the magnetite core. As a result, it can be concluded that the RMNPs have amine terminals suitable for further functionalization with DextCHO. To find how carboxylic acid reacts with iron (Fe), the bands related to the symmetrical and asymmetrical COO^−^ vibrations must clearly be shown, which are probably overlapped with 1628 and 1384 cm^−1^ bands. However, with a good approximation, the carboxylic acids made with unidentate complexes with the Fe metals exist in the structure of magnetite [51].

**Figure 2:**
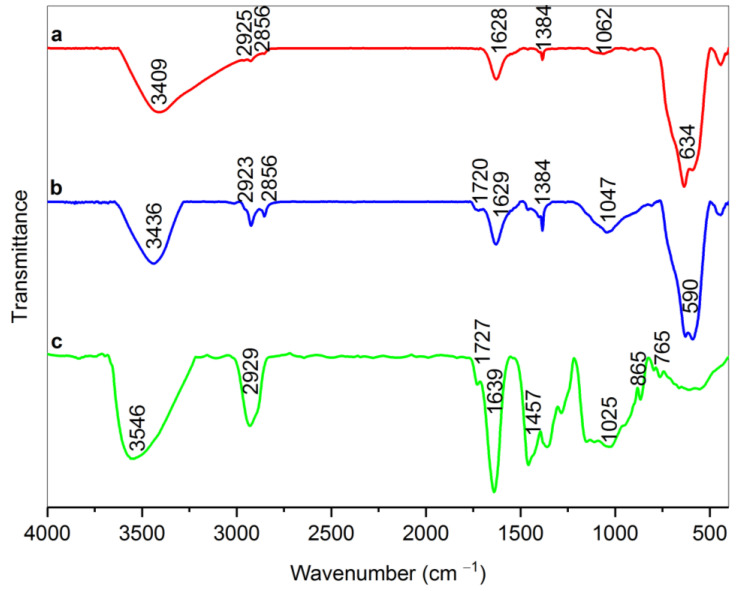
FT-IR spectra of RMNPs (a), Dext-RMNPs (b), and DextCHO (c).

According to Figure 2, it is obvious that there is a broad peak in the range of 1047 cm^−1^ in the dext-RMNPs spectrum (sample b) that can be attributed to the C–N stretching vibration. This finding can prove that Dext grafted on the arginine-modified magnetite NPs via C–N covalent bonds between the aldehyde groups of Dext and amine terminals of RMNPs [23,49]. It is also worth mentioning that the peaks related to the C–H stretching vibrations in the range of 2850–2930 cm^−1^ and C–H scissoring vibration at 1384 cm^−1^ exist due to the formation of new methylene groups (CH_2_) after that, the as-produced DextCHO-RMNPs has been reduced by sodium borohydride [49,50]. The peak in the 1720 cm^−1^ in dext-RMNPs is due to the C=O stretching vibration in the aldehyde group [52] and indicates that some aldehyde groups are still available on the DextCHO-RMNPs. These unreacted groups allow for conjugating various drugs, such as doxorubicin, to the synthesized DextCHO-RMNPs.

**Figure 3:**
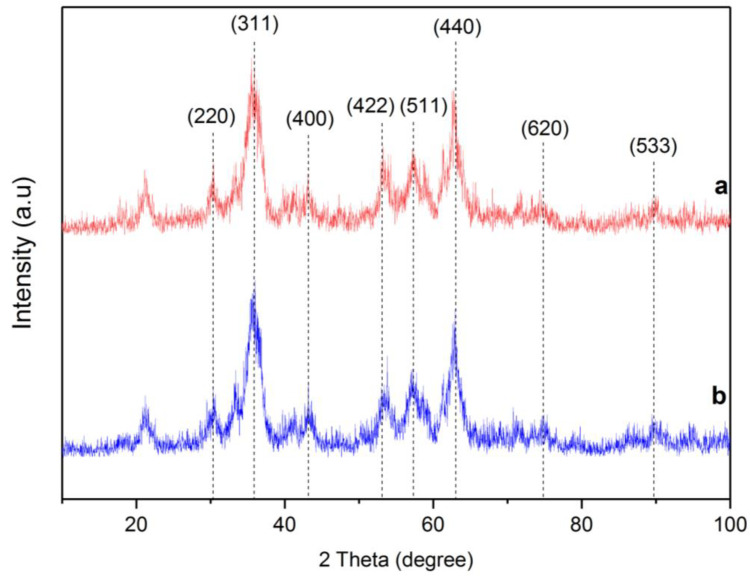
XRD pattern of RMNPs (a) and DextCHO-RMNPs (b).

The X-ray diffraction patterns of RMNPs and DextCHO-RMNPs are shown in Figure 3. A series of characteristic peaks, which are related to (220), (311), (400), (422), (511), (440), (620), and (533) crystallographic reflection planes at both RMNPs and DextCHO-RMNPs patterns are in good agreement with (JCPDS Card: 00019-0629), which belongs to the magnetite spinel structure [23,24,53]. The Scherrer equation estimates the crystallite size of RMNPs and DextCHO-RMNPs at 15.169 and 15.163 nm [23].

$$D=\frac{K\lambda }{\beta \;cos\theta }$$

where *λ* is the X-ray wavelength, *K* is a shape factor, which is about 0.9 for MNPs, *β* shows peak broadening at half the maximum peak intensity in radians, and *θ* is the Bragg angle. With a good approximation, it can be concluded that the surface modification cannot change the crystallite size, as previously observed [48]. The sizes of as-synthesized samples obtained from SEM are larger than the XRD pattern.

**Figure 4:**
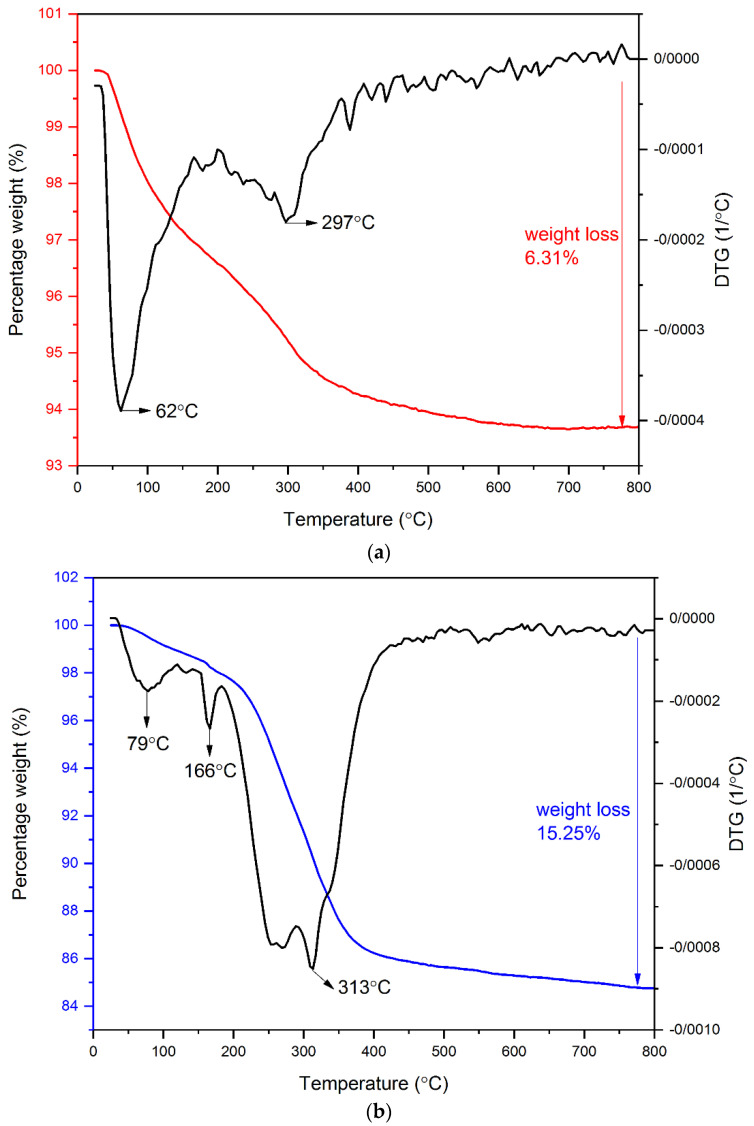
TG and DTG analysis of RMNPs (**a**), and DextCHO-RMNPs (**b**).

Figure 4 shows the TGA and the DTG curves of RMNPs and DextCHO-RMNPs. It can be observed that there are two stages of weight change in both samples. For RMNPs, the first stage of weight loss, which begins at 25 °C and continues to even 150˚C, could be attributed to the evaporation of water that is physically bounded to the samples [53,54]. The thermogram of RMNPs shows another stage of weight loss, which ranges from 200 °C to 600 °C, and matches well with the decomposition temperature range of pure arginine [55]. In the DextCHO-RMNPs curve, the main stage of the weight loss ranges between 220 °C and 400 °C. It continues to 800 °C, which corresponds to the decomposition of the polysaccharide Dext chain and is related to the processes such as dehydration, deamination, deacetylation, breaking of glycoside bonds, and pyranose ring opening [47,48,53,54]. Although this weight loss range could be associated with the overlapping decomposition of Dext and arginine, the total weight loss for DextCHO-RMNPs is larger than that in RMNPs. Therefore, it can be concluded that Dext was successfully coated on the RMNPs surface.

**Figure 5:**
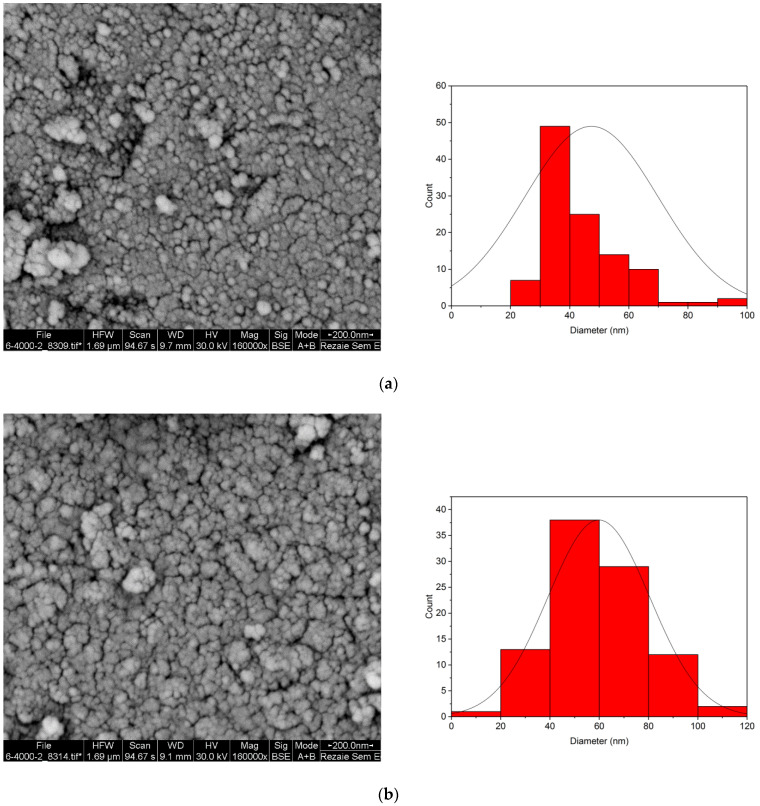
SEM image and size distribution of RMNPs (**a**) and DextCHO-RMNPs (**b**).

Figure 5 shows the SEM images of RMNPs and DextCHO-RMNPs, respectively. As these figures depicted, most MNPs have a spherical shape, and the average particle size is 47 and 59 nm in RMNPs and DextCHO-RMNPs, respectively. Therefore, increasing the particle size in DextCHO-RMNPs confirms that RMNPs have been coated with Dext successfully. In addition, particle size distribution becomes uniform in DextCHO-RMNPs in comparison with the RMNPs, according to the particle size distribution diagrams related to RMNPs and DextCHO-RMNPs.

**Figure 6:**
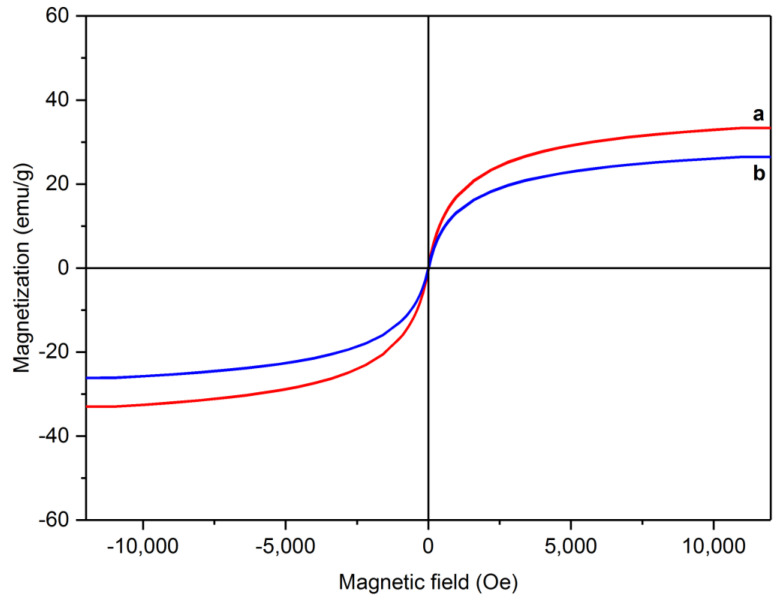
Magnetic hysteresis loops for RMNPs (a) and DextCHO-RMNPs (b).

To investigate the magnetic properties of the synthesized samples, the changes in magnetization as a function of magnetic field intensity were plotted. Figure 6 shows the hysteresis loops of RMNPs and DextCHO-RMNPs. They show superparamagnetic behavior since the coercivity and remanence in both samples are zero. By means of a reduction in the coercivity and limitation to zero, the anisotropy force is also reduced and becomes comparable with thermal energy, and consequently, it overcomes. The total magnetization can more easily change from one direction to the other even in the absence of the magnetic field [56]. As can be observed in Figure 6, the saturation magnetization of RMNPs and DextCHO-RMNPs are 33.32 and 26.45 emu g^−1^. The reduction in saturation magnetization might be due to the surrounding of RMNPs by a nonmagnetic Dext layer that proves that the aminated NPs were coated by Dext successfully. Moreover, the amount of saturation magnetization for aminated NPs is much less than the bare MNPs, which might be because of the Arg that has been located on the surface of NPs or disordered structure and incomplete crystallization at the surface during the synthesis [57].

Being functionalized by a layer of Dext, the RMNPs show longer circulation time in the blood and higher stability due to a lower degree of agglomeration, in which SEM images have observed, and the distribution size of the NPs have become more uniform and narrower after functionalizing with Dext [27,48]. These properties candidate the NPs to use them as a carrier for drug delivery systems and other biomedical applications, since various components with different functional groups could connect to the surface of these NPs because the reduction of agglomeration possibility and size uniformity could improve the efficiency of these NPs as drug carriers, due to fewer carriers lost by agglomeration and higher doses of therapeutic agents being uptook by target cells [58,59]. Moreover, coating the RMNPs with Dext makes them capable of being used as a contrast agent in MRI and magnetically mediated hyperthermia [27]. Besides, bonding the Dext to RMNPs through a chemical linkage, which is stronger than hydrogen bonding, keeps them more stable in biological media.

## 4. Conclusions

In this study, Dext was coated on MNPs, which were decorated by arginine previously, in a two-step co-precipitation method at an elevated temperature. Using Arg, Dext could be bonded to MNPs by chemical bonds, which are stronger than hydrogen bonding, and consequently, the stability of colloidal suspension in the water will increase. XRD analysis confirms that the core of synthesized samples is magnetite. To measure whether the Dext has bonded to the RMNPs or not, FT-IR and TGA-DTG were conducted on RMNPs and DextCHO-RMNPs. FT-IR spectra have demonstrated that Dext has been attached to the RMNPs through C-N chemical bonds. TGA-DTG curves have proved the results of FT-IR and shown stages of weight loss regarding the Dext and Arg decomposition, demonstrating that the RMNPs and DextCHO-RMNPs samples are successfully functionalized with Arg and Dext, respectively. To measure the magnetic properties, the VSM analysis has been performed, and it has been observed that MNPs show superparamagnetic behavior, which is necessary for targeting the drug delivery applications or other biomedical functions. The saturation magnetization is 33.32 and 26.45 emu g^−1^ for RMNPs and DextCHO-RMNPs, respectively. This decrease in magnetization confirms that Dext is coated on MNPs, and the reduction in magnetization is due to the covering of aminated core with a nonmagnetic layer. SEM images demonstrate that both synthesized samples consist of spherical NPs in which the distribution size for RMNPs is 47 nm and for the DextCHO-RMNPs is 59 nm. The existence of multiple hydroxyl groups on DextCHO-RMNPs helps it to bond to various functional groups or functional molecules such as proteins. It can be a subject for further studies on the effects of these MNPs on refolding as well as the denaturation processes of proteins.

## Data Availability

Data sharing is not applicable to this article as no datasets were generated or analyzed during the current study.

## References

[1] Gatoo M.A., Naseem S., Arfat M.Y., Mahmood Dar A., Qasim K., Zubair S. (2014). Physicochemical properties of nanomaterials: Implication in associated toxic manifestations. BioMed Res. Int..

[2] Khan I., Saeed K., Khan I. (2019). Nanoparticles: Properties, applications and toxicities. Arab. J. Chem..

[3] Lee J.E., Lee N., Kim T., Kim J., Hyeon T. (2011). Multifunctional mesoporous silica nanocomposite nanoparticles for theranostic applications. Acc. Chem. Res..

[4] Barrak H., Saied T., Chevallier P., Laroche G., M’nif A., Hamzaoui A.H. (2019). Synthesis, characterization, and functionalization of ZnO nanoparticles by N-(trimethoxysilylpropyl)ethylenediamine triacetic acid (TMSEDTA): Investigation of the interactions between Phloroglucinol and ZnO@ TMSEDTA. Arab. J. Chem..

[5] Mansha M., Qurashi A., Ullah N., Bakare F.O., Khan I., Yamani Z.H. (2016). Synthesis of In2O3/graphene heterostructure and their hydrogen gas sensing properties. Ceram. Int..

[6] Ganesh M., Hemalatha P., Peng M.M., Jang H.T. (2017). One pot synthesized Li, Zr doped porous silica nanoparticle for low temperature CO_2_ adsorption. Arab. J. Chem..

[7] He Y.P., Wang S.Q., Li C.R., Miao Y.M., Wu Z.Y., Zou B.S. (2005). Synthesis and characterization of functionalized silica-coated Fe3O4 superparamagnetic nanocrystals for biological applications. J. Phys. D Appl. Phys..

[8] Majewski P., Thierry B. (2008). Functionalized Magnetite Nanoparticles—Synthesis, Properties, and Bioapplications.

[9] Revia R.A., Zhang M. (2016). Magnetite nanoparticles for cancer diagnosis, treatment, and treatment monitoring: Recent advances. Mater. Today.

[10] Henderson E.D., Hua T., Kiran S., Khamis Z.I., Li Y., Sang QX A. (2022). Long-Term Effects of Nanoscale Magnetite on Human Forebrain-like Tissue Development in Stem-Cell-Derived Cortical Spheroids. ACS Biomater. Sci. Eng..

[11] Minaei S.E., Khoei S., Khoee S., Mahdavi S.R. (2022). Sensitization of glioblastoma cancer cells to radiotherapy and magnetic hyperthermia by targeted temozolomide-loaded magnetite tri-block copolymer nanoparticles as a nanotheranostic agent. Life Sci..

[12] Hsu C.H., Yu Y.S., Gu Y., Wu K.C. (2022). Modification of magnetite-doped NH2-MIL-100 (Fe) with aliphatic C8 carbon chain for feasible protein purification in reversed-phase mode. Sep. Purif. Technol..

[13] Vukadinović A., Milanović Z., Ognjanović M., Janković D., Radović M., Mirković M., Karageorgou M.A., Bouziotis P., Erić S., Vranješ-Đurić S. (2022). 90Y-CA/SPIONs for dual magnetic hyperthermia-radionuclide nanobrachytherapy of solid tumours. Nanotechnology.

[14] Włodarczyk A., Gorgoń S., Radoń A., Bajdak-Rusinek K. (2022). Magnetite Nanoparticles in Magnetic Hyperthermia and Cancer Therapies: Challenges and Perspectives. Nanomaterials.

[15] Wang X., Li C., Qian J., Lv X., Li H., Zou J., Zhang J., meng X., Liu H., Qian Y. (2021). NIR-II Responsive Hollow Magnetite Nanoclusters for Targeted Magnetic Resonance Imaging-Guided Photothermal/Chemo-Therapy and Chemodynamic Therapy. Small.

[16] Theerdhala S., Bahadur D., Vitta S., Perkas N., Zhong Z., Gedanken A. (2010). Sonochemical stabilization of ultrafine colloidal biocompatible magnetite nanoparticles using amino acid, L-arginine, for possible bio applications. Ultrason. Sonochem..

[17] Ebrahiminezhad A., Ghasemi Y., Rasoul-Amini S., Barar J., Davaran S. (2013). Preparation of novel magnetic fluorescent nanoparticles using amino acids. Colloids Surf. B Biointerfaces.

[18] Tapiero H., Mathe G., Couvreur P., Tew KD I. (2002). Arginine. Biomed. Pharmacother..

[19] Lee H.Y., Li Z., Chen K., Hsu A.R., Xu C., Xie J., Sun S., Chen X. (2008). PET/MRI dual-modality tumor imaging using arginine-glycine-aspartic (RGD)–conjugated radiolabeled iron oxide nanoparticles. J. Nucl. Med..

[20] Sattarahmady N., Azarpira N., Hosseinpour A., Heli H., Zare T. (2016). Albumin coated arginine-capped magnetite nanoparticles as a paclitaxel vehicle: Physicochemical characterizations and in vitro evaluation. J. Drug Deliv. Sci. Technol..

[21] Veiseh O., Kievit F.M., Mok H., Ayesh J., Clark C., Fang C., Leung M., Arami H., Park J.O., Zhang M. (2011). Cell transcytosing poly-arginine coated magnetic nanovector for safe and effective siRNA delivery. Biomaterials.

[22] Hristov D., McCartney F., Beirne J., Mahon E., Reid S., Bhattacharjee S., Penarier G., Werner U., Bazile D.V., Brayden D.J. (2019). Silica-coated nanoparticles with a core of zinc, L-arginine, and a peptide designed for oral delivery. ACS Appl. Mater. Interfaces.

[23] Bagherpour A.R., Kashanian F., Ebrahimi S.S., Habibi-Rezaei M. (2018). L-arginine modified magnetic nanoparticles: Green synthesis and characterization. Nanotechnology.

[24] Kashanian F., Habibi-Rezaei M., Bagherpour A.R., Seyedarabi A., Moosavi-Movahedi A.A. (2017). Magnetic nanoparticles as double-edged swords: Concentration-dependent ordering or disordering effects on lysozyme. RSC Adv..

[25] Kashanian F., Habibi-Rezaei M., Moosavi-Movahedi A.A., Bagherpour A.R., Vatani M. (2018). The ambivalent effect of Fe3O4 nanoparticles on the urea-induced unfolding and dilution-based refolding of lysozyme. Biomed. Mater..

[26] Alimohammadi V., Seyyed Ebrahimi S.A., Kashanian F., Lalegani Z., Habibi-Rezaei M., Hamawandi B. (2022). Hydrophobic Magnetite Nanoparticles for Bioseparation: Green Synthesis, Functionalization, and Characterization. Magnetochemistry.

[27] Hauser A.K., Mathias R., Anderson K.W., Hilt J.Z. (2015). The effects of synthesis method on the physical and chemical properties of dextran coated iron oxide nanoparticles. Mater. Chem. Phys..

[28] Sadighian S., Rostamizadeh K., Hosseini-Monfared H., Hamidi M. (2014). Doxorubicin-conjugated core–shell magnetite nanoparticles as dual-targeting carriers for anticancer drug delivery. Colloids Surf. B Biointerfaces.

[29] Mahdavi M., Ahmad M.B., Haron M.J., Namvar F., Nadi B., Rahman M.Z.A., Amin J. (2013). Synthesis, surface modification and characterisation of biocompatible magnetic iron oxide nanoparticles for biomedical applications. Molecules.

[30] Alaghmandfard A., Madaah Hosseini H.R. (2021). A facile, two-step synthesis and characterization of Fe_3_O_4_–LCysteine–graphene quantum dots as a multifunctional nanocomposite. Appl. Nanosci..

[31] Lajmorak A., Seyyed Ebrahimi S.A., Yazdian F., Lalegani Z., Hamawandi B. (2022). The Effect of Trehalose Coating for Magnetite Nanoparticles on Stability of Egg White Lysozyme. Int. J. Mol. Sci..

[32] Xu X.Q., Shen H., Xu J.R., Xu J., Li X.J., Xiong X.M. (2005). Core-shell structure and magnetic properties of magnetite magnetic fluids stabilized with dextran. Appl. Surf. Sci..

[33] Qu J., Liu G., Wang Y., Hong R. (2010). Preparation of Fe3O4–chitosan nanoparticles used for hyperthermia. Adv. Powder Technol..

[34] Barrera C., Herrera A.P., Rinaldi C. (2009). Colloidal dispersions of monodisperse magnetite nanoparticles modified with poly (ethylene glycol). J. Colloid Interface Sci..

[35] Haghighi A.H., Khorasani M.T., Faghih Z., Farjadian F. (2020). Effects of different quantities of antibody conjugated with magnetic nanoparticles on cell separation efficiency. Heliyon.

[36] Maingret V., Chartier C., Six J.L., Schmitt V., Héroguez V. (2022). Pickering emulsions stabilized by biodegradable dextran-based nanoparticles featuring enzyme responsiveness and co-encapsulation of actives. Carbohydr. Polym..

[37] Van Tomme S.R., Hennink W.E. (2007). Biodegradable dextran hydrogels for protein delivery applications. Expert Rev. Med. Devices.

[38] Su H., Jia Q., Shan S. (2016). Synthesis and characterization of Schiff base contained dextran microgels in water-in-oil inverse microemulsion. Carbohydr. Polym..

[39] Curcio M., Diaz-Gomez L., Cirillo G., Concheiro A., Iemma F., Alvarez-Lorenzo C. (2017). pH/redox dual-sensitive dextran nanogels for enhanced intracellular drug delivery. Eur. J. Pharm. Biopharm..

[40] Linh P.H., Phuc N.X., Hong L.V., Uyen L.L., Chien N.V., Nam P.H., Quy N.T., Nhung H.T.M., Phong P.T., Lee I.J. (2018). Dextran coated magnetite high susceptibility nanoparticles for hyperthermia applications. J. Magn. Magn. Mater..

[41] Shaterabadi Z., Nabiyouni G., Soleymani M. (2017). High impact of in situ dextran coating on biocompatibility, stability and magnetic properties of iron oxide nanoparticles. Mater. Sci. Eng. C.

[42] Chircov C., Ștefan R.E., Dolete G., Andrei A., Holban A.M., Oprea O.C., Vasile B.S., Neacșu I.A., Tihăuan B. (2022). Dextran-Coated Iron Oxide Nanoparticles Loaded with Curcumin for Antimicrobial Therapies. Pharmaceutics.

[43] Predoi G., Ciobanu C.S., Iconaru S.L., Predoi D., Dreghici D.B., Groza A., Barbuceanu F., Cimpeanu C., Badea M.-L., Barbuceanu S.F. (2021). Preparation and Characterization of Dextran Coated Iron Oxide Nanoparticles Thin Layers. Polymers.

[44] Gim S., Zhu Y., Seeberger P.H., Delbianco M. (2019). Carbohydrate-based nanomaterials for biomedical applications. Wiley Interdiscip. Rev. Nanomed. Nanobiotechnol..

[45] Gupta A.K., Gupta M. (2005). Cytotoxicity suppression and cellular uptake enhancement of surface modified magnetic nanoparticles. Biomaterials.

[46] Wada S., Tazawa K., Furuta I., Nagae H. (2003). Antitumor effect of new local hyperthermia using dextran magnetite complex in hamster tongue carcinoma. Oral Dis..

[47] Hong R.Y., Feng B., Chen L.L., Liu G.H., Li H.Z., Zheng Y., Wei D.G. (2008). Synthesis, characterization and MRI application of dextran-coated Fe3O4 magnetic nanoparticles. Biochem. Eng. J..

[48] Hong R.Y., Li J.H., Qu J.M., Chen L.L., Li H.Z. (2009). Preparation and characterization of magnetite/dextran nanocomposite used as a precursor of magnetic fluid. Chem. Eng. J..

[49] Stuart B.H. (2004). Infrared Spectroscopy: Fundamentals and Applications.

[50] Pavia D.L., Lampman G.M., Kriz G.S., Vyvyan J.A. (2014). Introduction to Spectroscopy.

[51] Nakamoto K. (2009). Infrared and Raman Spectra of Inorganic and Coordination Compounds, Part B: Applications in Coordination, Organometallic, and Bioinorganic Chemistry.

[52] Su H., Han X., He L., Deng L., Yu K., Jiang H., Wu C., Jia Q., Shan S. (2019). Synthesis and characterization of magnetic dextran nanogel doped with iron oxide nanoparticles as magnetic resonance imaging probe. Int. J. Biol. Macromol..

[53] Can H.K., Kavlak S., ParviziKhosroshahi S., Güner A. (2018). Preparation, characterization and dynamical mechanical properties of dextran-coated iron oxide nanoparticles (DIONPs). Artif. Cells Nanomed. Biotechnol..

[54] Barbosa-Barros L., García-Jimeno S., Estelrich J. (2014). Formation and characterization of biobased magnetic nanoparticles double coated with dextran and chitosan by layer-by-layer deposition. Colloids Surf. A Physicochem. Eng. Asp..

[55] Wang Z., Zhu H., Wang X., Yang F., Yang X. (2009). One-pot green synthesis of biocompatible arginine-stabilized magnetic nanoparticles. Nanotechnology.

[56] Spaldin N.A. (2010). Magnetic Materials: Fundamentals and Applications.

[57] Jiang Q.L., Zheng S.W., Hong R.Y., Deng S.M., Guo L., Hu R.L., Gao B., Huang M., Cheng L.F., Liu G.H. (2014). Folic acid-conjugated Fe3O4 magnetic nanoparticles for hyperthermia and MRI in vitro and in vivo. Appl. Surf. Sci..

[58] Akbarzadeh A., Samiei M., Davaran S. (2012). Magnetic nanoparticles: Preparation, physical properties, and applications in biomedicine. Nanoscale Res. Lett..

[59] Plank C., Zelphati O., Mykhaylyk O. (2011). Magnetically enhanced nucleic acid delivery. Ten years of magnetofection—Progress and prospects. Adv. Drug Deliv. Rev..

